# Effects of Oral Collagen for Skin Anti-Aging: A Systematic Review and Meta-Analysis

**DOI:** 10.3390/nu15092080

**Published:** 2023-04-26

**Authors:** Szu-Yu Pu, Ya-Li Huang, Chi-Ming Pu, Yi-No Kang, Khanh Dinh Hoang, Kee-Hsin Chen, Chiehfeng Chen

**Affiliations:** 1 School of Medicine, College of Medicine, Taipei Medical University, Taipei City 110, Taiwan; b101110047@tmu.edu.tw; 2 Department of Public Health, School of Medicine, College of Medicine, Taipei Medical University, Taipei City 11031, Taiwan; ylhuang@tmu.edu.tw; 3 Division of Plastic Surgery, Department of Surgery, Cathay General Hospital, Taipei City 106, Taiwan; pkman9335@msn.com; 4 School of Medicine, College of Life Science and Medicine, National Tsing Hua University, Hsinchu City 300, Taiwan; 5 Cochrane Taiwan, Taipei Medical University, Taipei City 110, Taiwan; kynacad@gmail.com (Y.-N.K.); keehsin@tmu.edu.tw (K.-H.C.); 6 Evidence-Based Medicine Center, Wan Fang Hospital, Taipei Medical University, Taipei City 116, Taiwan; 7 Research Center of Big Data and Meta-Analysis, Wan Fang Hospital, Taipei Medical University, Taipei City 116079, Taiwan; 8 Institute of Health Policy and Management, College of Public Health, National Taiwan University, Taipei City 100, Taiwan; 9 Department of Histopathology, Hai Phong University of Medicine and Pharmacy, Hai Phong 04254, Vietnam; hdkhanh@hpmu.edu.vn; 10 Post-Baccalaureate Program in Nursing, College of Nursing, Taipei Medical University, Taipei City 11031, Taiwan; 11 Department of Nursing, Wan Fang Hospital, Taipei Medical University, Taipei City 11696, Taiwan; 12 Research Center in Nursing Clinical Practice, Wan Fang Hospital, Taipei Medical University, Taipei 11696, Taiwan; 13 Evidence-Based Knowledge Translation Center, Wan Fang Hospital, Taipei Medical University, Taipei City 11696, Taiwan; 14 School of Medicine, Faculty of Health and Medical Sciences, Taylor’s University, Selangor 47500, Malaysia; 15 Division of Plastic Surgery, Department of Surgery, Wan Fang Hospital, Taipei Medical University, Taipei City 116, Taiwan

**Keywords:** oral collagen, skin, anti-aging, systematic review, meta-analysis

## Abstract

This paper presents a systematic review and meta-analysis of 26 randomized controlled trials (RCTs) involving 1721 patients to assess the effects of hydrolyzed collagen (HC) supplementation on skin hydration and elasticity. The results showed that HC supplementation significantly improved skin hydration (test for overall effect: Z = 4.94, *p* < 0.00001) and elasticity (test for overall effect: Z = 4.49, *p* < 0.00001) compared to the placebo group. Subgroup analyses demonstrated that the effects of HC supplementation on skin hydration varied based on the source of collagen and the duration of supplementation. However, there were no significant differences in the effects of different sources (*p* = 0.21) of collagen or corresponding measurements (*p* = 0.06) on skin elasticity. The study also identified several biases in the included RCTs. Overall, the findings suggest that HC supplementation can have positive effects on skin health, but further large-scale randomized control trials are necessary to confirm these findings.

## 1. Introduction

The skin, the largest organ of the body exposed to the external environment, is affected by both intrinsic and extrinsic factors in the aging process [1]. Skin aging is characterized by dehydration, a loss of skin elasticity, and the presence of wrinkles [2]. Skin aging has attracted considerable attention because of the increasingly high beauty standards. Because many countries are becoming aging societies, the psychosocial effects of skin aging increases the need for effective interventions [3]. In this context, the use of nutraceuticals as supplements has increased in recent years [4].

Collagen is the main protein structure of various connective tissues, which constitutes 80% of the dry weight of human skin [5]. Collagen is characterized by a triple helix structure formed by the repetition of glycine every third residue, and particularly by proline and hydroxyproline in the other residues [6]. Collagen, the most prevalent component of extracellular matrix, provides mechanical support and directs tissue development [7].

Aging induces a decline in the enzymes involved in the post-translational processing of collagen, reducing the number of fibroblasts that synthesize collagen and vessels that supply the skin [8]. The decline in skin quality with age is characterized by a reduction in collagen synthesis and a decrease in skin vascularity, leading to decreased elasticity and the formation of wrinkles [9]. These changes are due to the decline in fibroblast activity and a decrease in the number of blood vessels in the skin [10]. Therefore, the skin undergoes regressive changes with age such as dehydration, a loss of elasticity, and a reduction in epidermal thickness [11]. Various nutrients and supplements are used to improve skin health and maintain a youthful skin appearance [12]. These strategies include topical creams, injectable fillers, and collagen supplements. Topical creams contain collagen as one of the ingredients, and they are designed to enhance skin hydration and firmness [13]. However, topical creams have limited ability to penetrate the skin, which can reduce their effectiveness [13]. Injectable fillers such as hyaluronic acid fillers, stimulate collagen production and provide immediate results by plumping the skin [14]. However, they can be expensive and come with the risk of adverse events such as bruising, swelling, and infection [14]. On the other hand, collagen supplements, particularly those containing hydrolyzed collagen peptides, have been shown to be safe and cost-effective compared to other collagen-based strategies. Furthermore, collagen supplements have the advantage of being taken orally, making them easy to incorporate into daily routines [15].

Among these supplements, hydrolyzed collagen (HC) is the most popular and promising skin anti-aging nutraceutical [16]. Other studies have indicated that alanine–hydroxyproline–glycine and serine–hydroxyproline–glycine can be detected in human blood 1 h after the oral ingestion of HC [17,18] and deposited on the skin [19].

A recent study demonstrated that HC improves skin hydration and elasticity [16]. Nevertheless, not all sources of HC have the same efficacy. Even at the same dose and duration of administration, some specific sources of collagens are more effective than others [20]. Therefore, studies are required to determine the proper source and therapeutic duration of HC against skin aging.

Because an increasing number of clinical studies on collagen supplements have been conducted globally, their results must be summarized in a systematic review and meta-analysis. Therefore, this systematic review and meta-analysis investigated the effects of collagen supplementation on skin hydration and elasticity.

## 2. Materials and Methods

### 2.1. Search Strategy, Inclusion Criteria, and Exclusion Criteria

We performed a literature search in the Embase, PubMed, and Cochrane Library databases by using the following search terms from Medical Subject Headings with no restrictions applied: (collagen OR hydrolyzed collagen) AND (anti-aging). Relevant studies published before December 2022 were identified. We included studies that met the following criteria: (1) applying a randomized clinical trial (RCT) design; (2) including healthy adults (aged ≥ 18 years); (3) including patients who received HC; (4) being full-text articles written in English. We excluded studies that (1) assessed the combined effect of collagen supplement with another supplement or (2) were RCTs that were not written in English. We extracted raw data from the graphs in articles using WebPlotDigitizer [21].

### 2.2. Data Extraction

Two independent reviewers (S-YP, CC) extracted the basic information of the included studies. The following types of information were extracted: study meta-data (i.e., first author, publication year, and study design) and information on the study sample (i.e., number of patients, gender, mean age, and baseline characteristics of the treatment and placebo groups), intervention (i.e., the dose of collagen supplement and form), and outcomes (i.e., hydration and elasticity). Continuous outcomes are presented in terms of the mean ± standard deviation (SD), and discrete data are presented in terms of percentage.

### 2.3. Statistical Analysis, Sensitivity Analysis and Bias Assessment

We used a random-effects model to calculate the SD and mean difference of the identified studies. A *p* value of <0.05 indicated statistical significance. The levels of heterogeneity among the included studies were determined using Hedge’s I^2^ tests, and forest plots were generated for each included study. Moreover, I^2^ ≥ 50% indicated high heterogeneity [22]. The general effect test result was reported as a z-value, which supported the inference of the 95% confidence interval (CI). A sensitivity analysis was performed to negate the effect of potentially influential studies. Each study was classified in accordance with the Cochrane Handbook for Systematic Reviews of Interventions [23]. The Cochrane risk of bias (RoB) 2.0 tool was used to assess the risk of bias in the included RCTs. Five domains of bias were evaluated (selection, performance, detection, attrition, and reporting bias) [24]. In this meta-analysis, all outcomes were analyzed using RevMan software (version 5.4).

## 3. Results

### 3.1. Research Results and Study Characteristics

**Figure 1:**
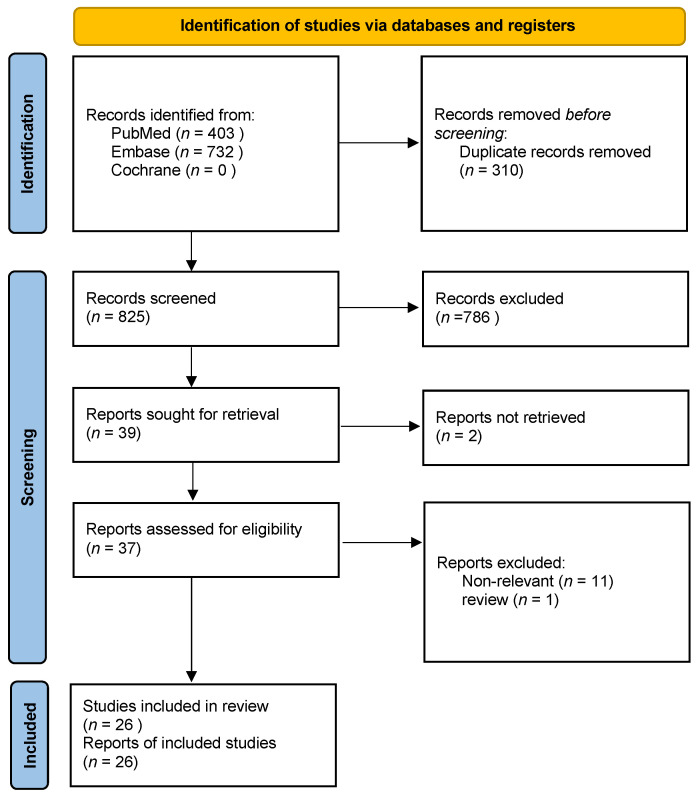
Flowchart of the systematic review and meta-analysis according to the PRISMA guidelines.

Figure 1 shows the flowchart of the literature search process performed in accordance with the Preferred Reporting Items for Systematic Reviews and Meta-Analyses guidelines [25]. We identified 1135 studies in our initial search. After removing duplicates and screening titles or abstracts of related articles, we assessed the full-text articles of the remaining 37 studies. Of these studies, 26 articles were included in this systematic review and meta-analysis.

### 3.2. Study Characteristics

A total of 26 RCTs involving 1721 patients were included in this meta-analysis. The duration of the HC supplementation of the included studies ranged from 2 to 12 weeks. Among the included RCTs, 14 focused on collagens extracted from fish, one focused on collagens extracted from bovine, one focused on collagens extracted from chicken, two focused on collagens extracted from porcine, and nine lacked information regarding the source of collagen. The study characteristics of the included RCTs are presented in Table 1.

The measurement of skin hydration levels is commonly conducted using a non-invasive tool called a corneometer. This instrument emits a high-frequency electric current into the skin’s surface and measures the amount of water present in the top layer, expressed in corneometry units. The corneometer is widely used in evaluating the effectiveness of topical products and assessing overall skin health by providing valuable insights into the skin’s moisture barrier. Therefore, it is considered as a valuable tool in measuring the skin hydration levels and assessing the efficacy of skincare products [18,26,27,28,29,30,31,32,33]. On the other hand, the measurement of skin elasticity is often conducted using cutometry, a non-invasive technique that provides valuable insights into skin health. It works by applying a controlled negative pressure to a small area of the skin and measuring the resulting deformation, which is directly proportional to the skin’s elasticity. Cutometry is widely used in research and clinical settings to assess the skin elasticity levels and monitor changes in the skin over time. Overall, it is a safe and reliable tool for evaluating skin health [18,26,27,29,32,33,34,35,36,37].

**Table 1:** Characteristics of the patients in the included studies.

Author (Year)	Female/Male	Age Range	Time (Weeks)	Intervention (Origin)	Outcome Extracted
Proksch et al. (2014a) [18]	60/0	35–55	8, 12	2.5 g HC/5 g HC (porcine)	Elasticity/hydration/trans-epidermal water loss (TEWL)/wrinkles
Proksch et al. (2014b) [38]	107/0	45–65	8, 12	2.5 g collagen peptides	Wrinkles/biopsy/procollagen type/elastin/fibrillin
Yoon et al. (2014) [39]	44/0	>44	12	3 g HC (fish)	Procollagen type 1/fibrillin 1/metalloproteinases 1 and 12/biopsies/immunohistochemical staining
Di Cerbo et al. (2014) [40]	30/0	40–45	4.5	372 mg HC	Cutaneous pH/hydration/sebum/elasticity/skin tone/elastin/elastase 2/fibronectin/hyaluronic acid/carbonyl proteins
Choi et al. (2014) [32]	24/8	30–48	5	3 g collagen peptides	Skin hydration/elasticity/TEWL/erythema/satisfaction questionnaire
Sugihara, Inoue, and Wang (2015) [28]	53/0	35–55	8	2.5 g HC (fish)	Hydration/elasticity/wrinkles
Campos et al. (2015) [29]	60/0	40–50	12	10 g HC	Corneal stratum hydration/skin viscoelasticity/dermal echogenicity/high-resolution photography
Asserin et al. (2015) [30]	134/0	40–65	8, 12	10 g HC (porcine)/10 g HC (fish)	Skin moisture/TEWL/dermal density/dermal echogenicity/dermal collagen fragmentation
Inoue, Sugihara, and Wang (2016) [34]	80/0	35–55	8	2.5 g collagen peptides	Skin moisture/elasticity/wrinkles
Genovese, Corbo, and Sibilla (2017) [41]	111/9	40–60	12	5 g HC	Elasticity/biopsies/subjective questionnaire
Koizumi et al. (2017) [27]	71/0	30–60	12	3 g collagen peptides	Wrinkles/moisture/elasticity/blood tests (γ-glutamyltransferase, mean corpuscular hemoglobin concentration, mean corpuscular hemoglobin, mean corpuscular volume, red blood cell, platelet, white blood cell, bilirubin, creatinine, total cholesterol, glucose, hemoglobin, hematocrit, alanine aminotransferase, aspartate aminotransferase, total protein and albumin)
Czajka et al. (2018) [42]	120/0	21–70	12	4 g HC	Elasticity/biopsies/self-perception questionnaire
Kim (2018) [43]	70/0	40–60	12	1000 mg collagen (fish)	Skin hydration/wrinkling/elasticity
Ito, Seki, and Ueda (2018) [31]	17/4	30–50	8	10 g collagen peptides (fish)	Elasticity/moisture/TEWL/skin pH/spots/wrinkle/skin pores/texture/density/collagen score/growth hormone (GH), insulin-like growth factor-1 (IGF-1)
Bolke et al. (2019) [26]	72/0	>35	12, 16	2.5 g collagen peptides	Hydration/elasticity/wrinkles/skin density/subjective questionnaire
Schwartz et al. (2019) [35]	113/0	36–59	12	0.6 g HC (chicken)	Erythema/hydration/TEWL/elasticity/wrinkles/dermal collagen/subjective questionnaire
Zmitek et al. (2020) [44]	31/0	40–65	12	4 g HC (fish)	Dermal density and thickness/viscoelasticity/hydration/TEWL/wrinkles/moisture/dermal microrelief
Laing et al. (2020) [45]	60/0	40–70	12	2.5 g collagen peptides	Dermal collagen fragmentation/subjective questionnaire
Sangsuwan and Asawanonda (2020) [37]	36/0	50–60	4, 8	5 g HC	Elasticity
Nomoto and Iizaka (2020) [33]	27/12	>65	8	12 g collagen peptides	Stratum corneum hydration/elasticity
Ping (2020) [46]	50/0	35–50	8	5.5 g collagen (fish)	Skin hydration/brightness/texture/crow’s feet/collagen content
Evans (2020) [47]	50/0	45–60	12	10 g HC (fish)	Wrinkles/elasticity/self-reported appearance
Tak (2021) [48]	84/0	40–60	12	1000 mg collagen tripeptides	Hydration/elasticity/wrinkles
Miyanaga (2021) [49]	99/0	35–50	12	1 g HC/5 g HC	Skin water content/TEWL/elasticity/thickness
Jung (2021) [50]	25/25	35–60	12	1000 mg collagen (fish)	Skin hydration/TEWL/texture/flexibility
Bianchi (2022) [51]	52/0	40–60	8	5 g HC	Skin moisturization/elasticity/wrinkle depth

### 3.3. Meta-Analysis Results

#### 3.3.1. Pooled Analysis of Selected Studies

Some articles were excluded from the research due to various reasons. Studies conducted by Campos, Czajka, Genovese, and Sangsuwan were not considered as they did not measure the hydration levels, which was a key parameter of interest. Similarly, the Asserin study did not measure elasticity, so its results could not be used to evaluate the impact of elasticity on the outcome measures. The Bianchi and Ping study was excluded due to the lack of standard deviation data for the placebo group, which was necessary for the statistical analysis. The Laing study did not provide sufficient direct data on moisture and elasticity, the primary outcomes of interest, and the provided microscopic observations and questionnaires were insufficient for the research. Finally, the Proksch study did not provide data for the placebo group, making it impossible to compare the results with those of the intervention group. Therefore, these studies did not meet the necessary criteria for inclusion in the research.

**Figure 2:**
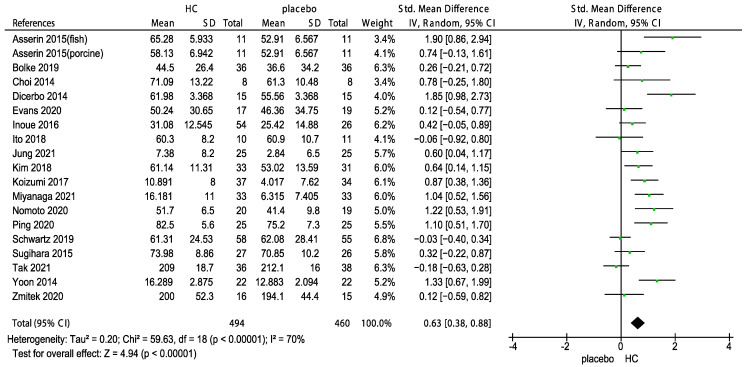
Forest plot of the included studies evaluating skin hydration in patients supplemented with HC and patients in the placebo group [26,27,28,30,31,32,33,34,35,39,40,43,44,46,47,48,49,50]. (HC: hydrolyzed collagen, CI: confidence intervals, SD: standard deviation, I^2^: heterogeneity).

**Figure 3:**
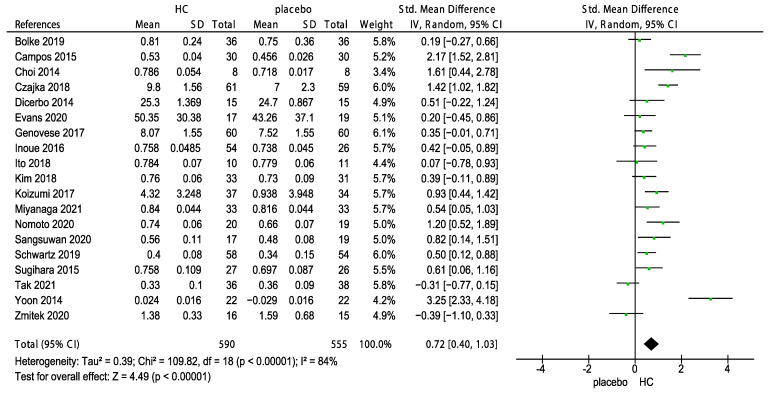
Forest plot of the included studies evaluating skin elasticity in patients supplemented with HC and patients in the placebo group [26,27,28,29,31,32,33,34,35,37,39,40,41,42,43,44,47,48,49]. (HC: hydrolyzed collagen, CI: confidence intervals, SD: standard deviation, I^2^: heterogeneity).

All included RCTs divided the patients into two groups according to the collagen measurement and skin hydration or elasticity, and then subjected to a meta-analysis. The standard mean difference (SMD) of 18 studies on the effects of HC and the placebo on skin hydration are shown in Figure 2. The overall pooled effect size of 0.63 (95% CI 0.38, 0.88) indicated that HC supplementation significantly improved skin hydration (z = 4.94, *p* < 0.00001). Figure 3 shows the forest plot of the meta-analysis of 19 studies on the effects of HC on skin elasticity; the results indicate that HC supplementation significantly improved skin elasticity (z = 4.49, *p* < 0.00001) compared with the placebo group at a pooled effect size of 0.72 (95% CI 0.40, 1.03).

#### 3.3.2. Subgroup Analysis

**Figure 4:**
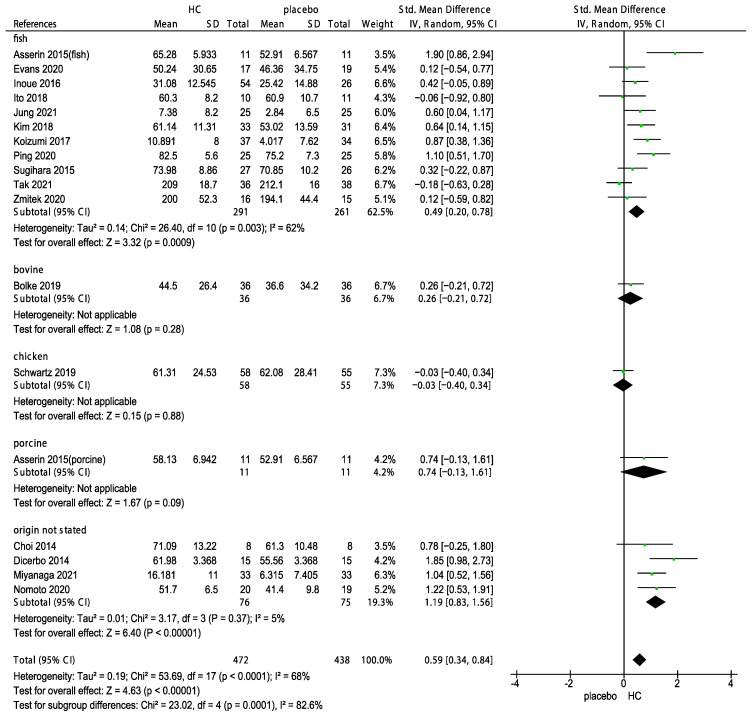
Forest plot for the subgroup analysis of skin hydration expressed as HC originating from fish, bovine, chicken, porcine, and unknown source in patients supplemented with HC and patients in the placebo group [26,27,28,30,31,32,33,34,35,40,43,44,46,47,48,49,50]. (HC: hydrolyzed collagen, CI: confidence intervals, SD: standard deviation, I^2^: heterogeneity).

**Figure 5:**
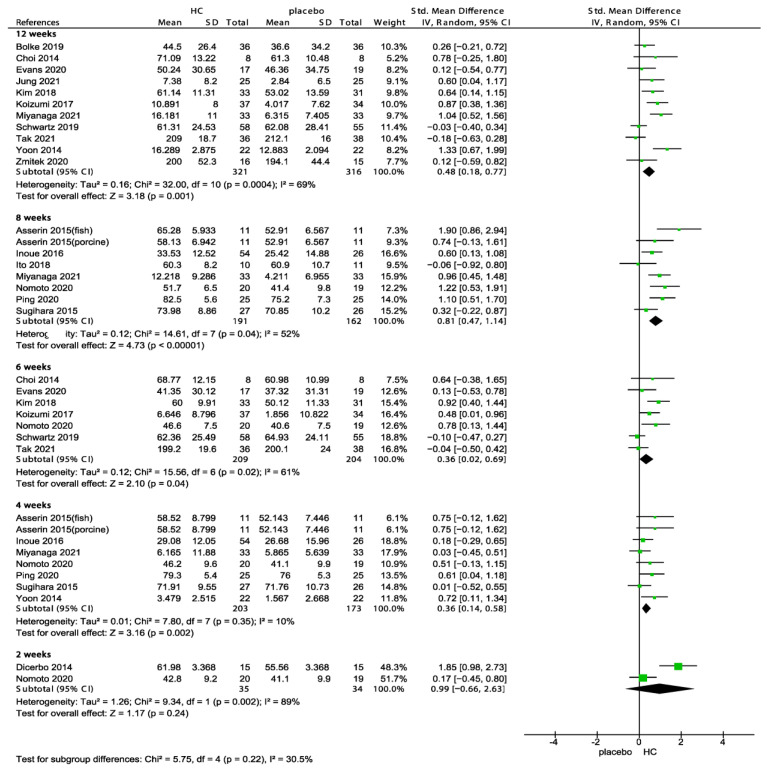
Forest plot for the subgroup analysis of skin hydration expressed as 2, 4, 6, 8, and 12 weeks in patients supplemented with HC and patients in the placebo group [26,27,28,29,30,31,32,33,34,35,39,40,43,44,46,47,48,49,50]. (HC: hydrolyzed collagen, CI: confidence intervals, SD: standard deviation, I^2^: heterogeneity).

**Figure 6:**
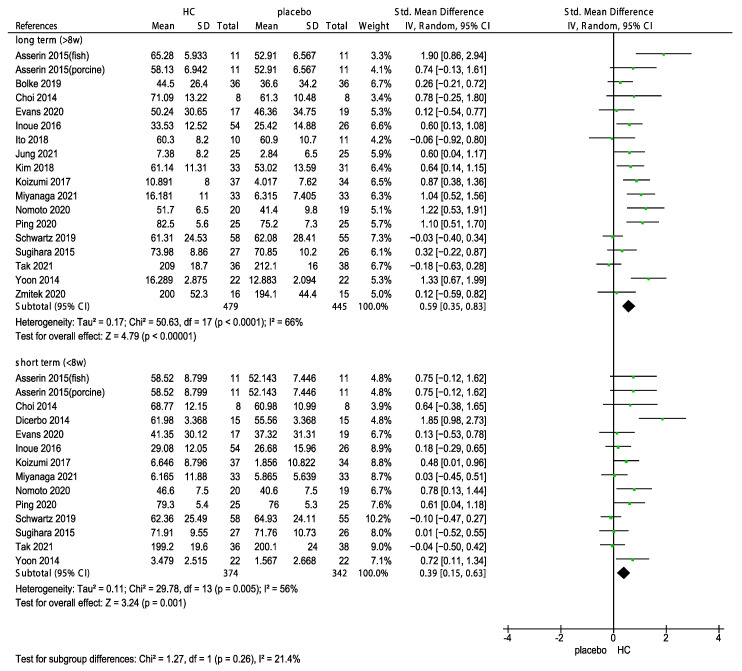
Forest plot for the subgroup analysis of skin hydration expressed as long-term (>8 weeks) and short-term (<8 weeks) in patients supplemented with HC and patients in the placebo group [26,27,28,30,31,32,33,34,35,39,40,43,44,46,47,48,49,50]. (HC: hydrolyzed collagen, CI: confidence intervals, SD: standard deviation, I^2^: heterogeneity).

Collagen supplements are available in various forms including gels, liquids, and capsules. The type of collagen used in these supplements can vary depending on the source, with some of the most common types including fish, porcine, chicken, and bovine collagen. A subgroup analysis was performed to determine the effects of multiple sources of HC supplements and duration on skin hydration. The results showed that the supplementation with HC originating from fish, bovine, chicken, porcine, and unknown source significantly improved skin hydration (Figure 4, *p* < 0.00001). Of these sources, HC originating from chicken had the weakest effect (−0.03, 95% CI −0.40, 0.34) on skin hydration. In addition, we performed subgroup analyses on the duration of HC supplementation for 2, 4, 6, 8, and 12 weeks. The forest plot analysis revealed that the effects of HC supplementation during 4 (*p* = 0.002), 6 (*p* = 0.04), 8 (*p* < 0.00001), and 12 weeks (*p* = 0.001) significantly differed, as shown in Figure 5. In addition, the effects of the long-term use (>8 weeks) of HC (0.59, 95% CI 0.35, 0.83) were more favorable than that of the short-term use (<8 weeks) of HC (0.39, 95% CI 0.15, 0.63, Figure 6).

**Figure 7:**
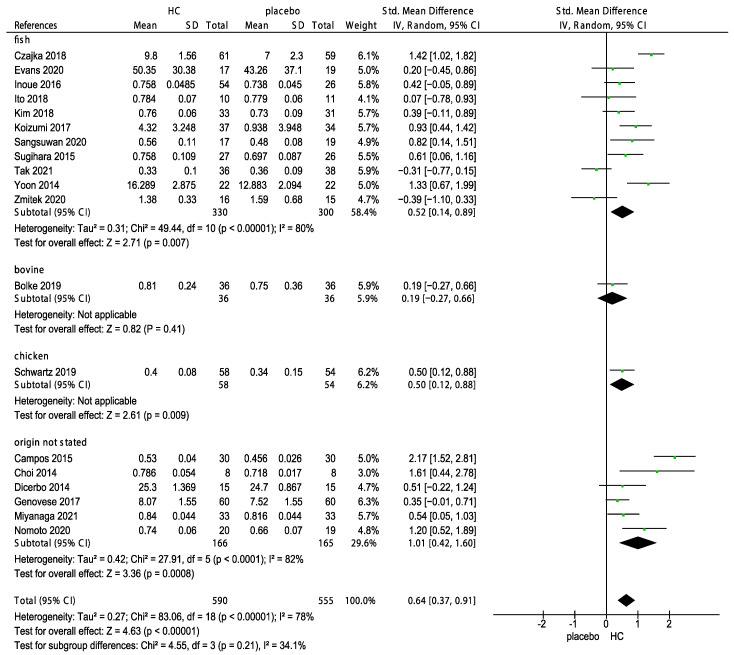
Forest plot for the subgroup analysis of skin elasticity expressed as HC originating from fish, bovine, chicken, porcine, and unknown source in patients supplemented with HC and patients in the placebo group [26,27,28,29,31,32,33,34,35,37,39,40,41,42,43,44,47,48,49]. (HC: hydrolyzed collagen, CI: confidence intervals, SD: standard deviation, I^2^: heterogeneity).

**Figure 8:**
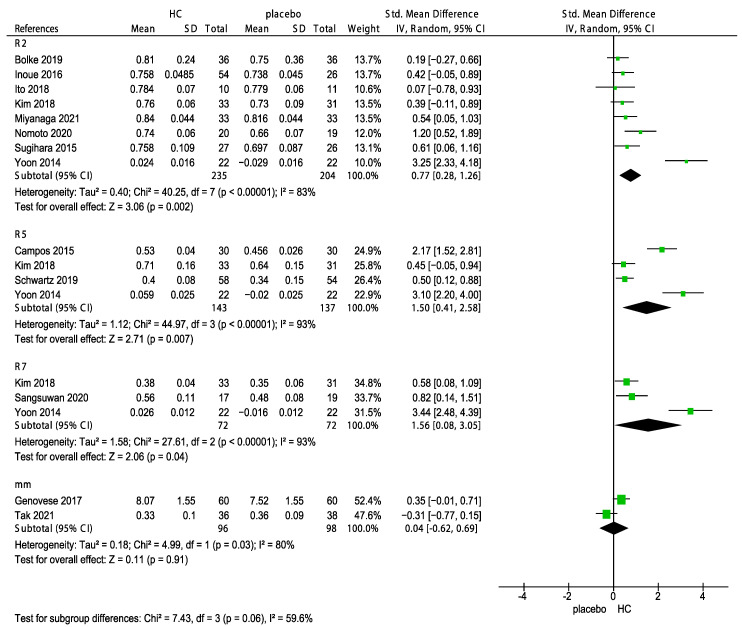
Forest plot for the subgroup analysis of skin elasticity expressed as R2 (Gross elasticity), R5 (Net elasticity; elastic portion of relaxation/elastic portion of suction), R7 (Elastic portion; elastic portion of relaxation/first maximum amplitude after suction), and mm in patients supplemented with hydrolyzed collagen (HC) and patients in the placebo group [26,28,29,31,33,34,35,37,39,41,43,48,49]. (HC: hydrolyzed collagen, CI: confidence intervals, SD: standard deviation, I^2^: heterogeneity).

**Figure 9:**
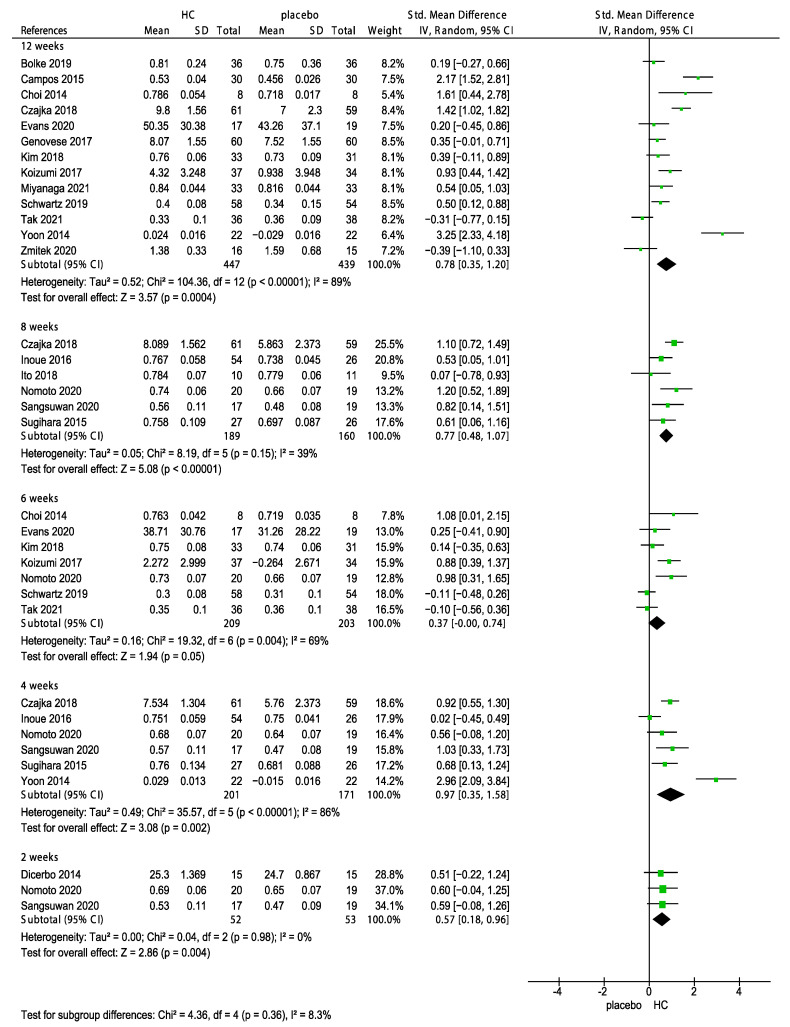
Forest plot for the subgroup analysis of skin elasticity expressed as 2, 4, 6, 8, and 12 weeks in patients supplemented with HC and patients in the placebo group [26,27,28,29,31,32,33,34,35,37,39,40,41,42,43,44,47,48,49]. (HC: hydrolyzed collagen, CI: confidence intervals, SD: standard deviation, I^2^: heterogeneity).

**Figure 10:**
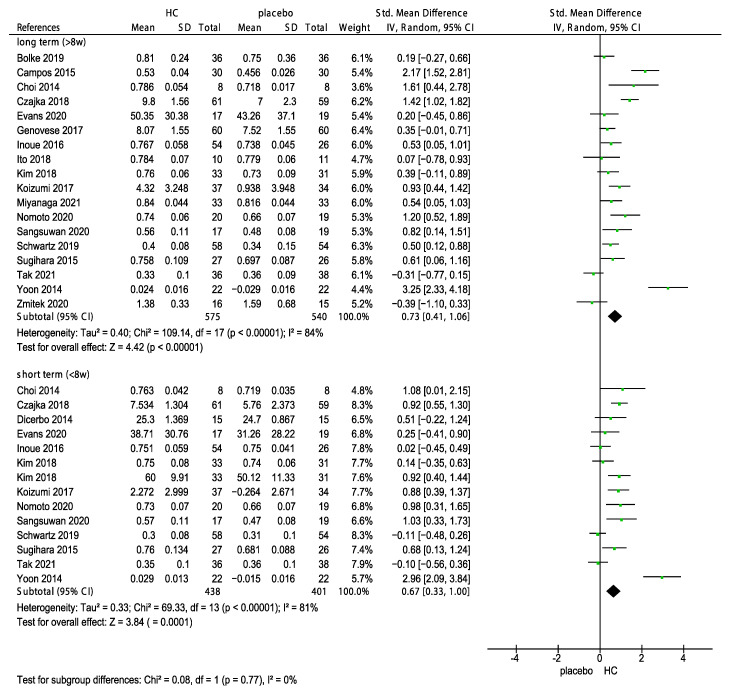
Forest plot for the subgroup analysis of skin elasticity expressed as long-term (>8 weeks) and short-term (<8 weeks) in patients supplemented with HC and patients in the placebo group [26,27,28,29,31,32,33,34,35,37,39,40,41,42,43,44,47,48,49]. (HC: hydrolyzed collagen, CI: confidence intervals, SD: standard deviation, I^2^: heterogeneity).

In addition, three subgroup analyses of the effects of sources of HC, corresponding measurements (R2: Gross elasticity, R5: Net elasticity; elastic portion of relaxation/elastic portion of suction, R7: Elastic portion; elastic portion of relaxation/first maximum amplitude after suction and mm by cutometer) and the duration of HC supplementation on skin elasticity were performed. The subgroup analyses indicated no significant differences in the effects of various sources of HC (*p* = 0.21, Figure 7) and the corresponding measurements (*p* = 0.06, Figure 8) on skin elasticity. The subgroup analysis on the duration revealed that 6 weeks of HC supplementation showed no positive effect on skin elasticity (*p* = 0.05, Figure 9). Furthermore, the effect of the long-term use (>8 weeks) of HC (0.73, 95% CI 0.41, 1.06) was more favorable than that of the short-term use (<8 weeks) of HC (0.67, 95% CI 0.33, 1.00) on skin elasticity. The results of the subgroup analyses are presented in Figure 10.

### 3.4. Bias

**Figure 11:**
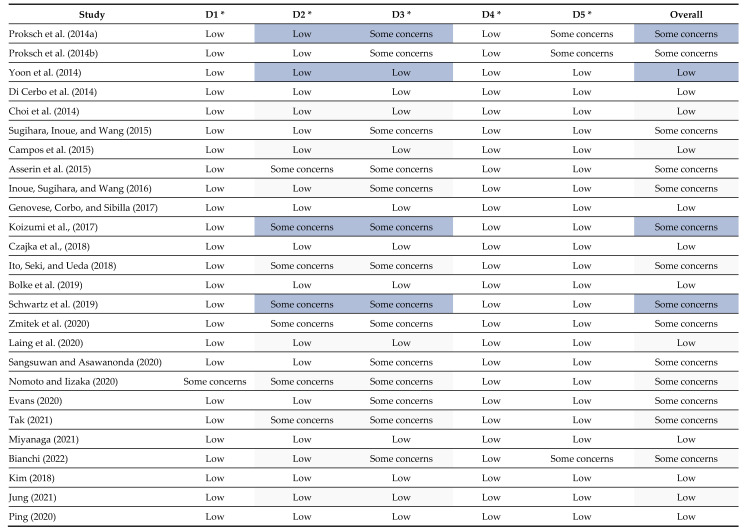
Risk of bias [18,26,27,28,29,30,31,32,33,34,35,37,38,39,40,41,42,43,44,45,46,47,48,49,50,51]. * D1: Randomization process; D2: Deviations from the intended interventions; D3: Missing outcome data; D4: Measurement of outcome; D5: Selection of the reported result.

In conducting systematic reviews and meta-analyses, it is important to examine the quality of research studies and potential biases. One common method for assessing bias is through the use of RoB (Risk of Bias). RoB evaluates various aspects of a study that could lead to bias such as incomplete outcome data and selective outcome reporting. Each aspect is evaluated based on predefined criteria, and an overall assessment of the study’s risk of bias is made. The goal of RoB is to provide an impartial evaluation of the study’s design, implementation, and reporting to aid in determining the study’s reliability and suitability for inclusion in systematic reviews or meta-analyses [24]. At the study level, we found an RoB in the bias arising from the randomization process in one study [33], bias due to deviations from intended intervention in seven studies [27,30,31,33,35,44,48], bias due to missing outcome data in thirteen studies [18,27,28,30,31,33,34,35,37,44,47,48,51], and bias in the selection of the reported results in two studies [18,51]. Figure 11 provides additional details on the RoB assessment results for the included RCTs.

## 4. Discussion

To evaluate the effects of collagen supplements on skin aging, we analyzed 26 RCTs to assess the efficacy of oral collagen supplements on skin hydration and elasticity, both of which characterize skin aging. The trials measured skin hydration and elasticity on various areas of the body including the cheek, forearm, and forehead. By analyzing these parameters, our findings revealed that oral collagen supplements improved skin hydration and elasticity. The beneficial effects were significant after 8 weeks or more of HC supplementation.

### 4.1. Hydration

The key molecule involved in skin moisture is hyaluronic acid, a glycosaminoglycan with a unique capacity to retain water molecules [52]. The most striking histochemical change observed in aging skin is the gradual loss of epidermal hyaluronic acid [53]. Oral administration of collagen hydrolysates include rich proline-hydroxyproline, which stimulates hyaluronic acid production in the dermal fibroblast cells [54].

Our study findings revealed that supplementation with oral collagens improved skin hydration, which is consistent with previous findings. Cao et al. reported that the concentration of moisture in the skin of mice treated with collagen peptides (CPs) was significantly higher compared with that of the control mice (*p* < 0.05) [55]. Sun et al. revealed that collagen as a single supplement showed remarkable effects on skin hydration, with an SMD of 0.77 (95% CI 0.60, 0.94; *p* < 0.00001) compared with a placebo [56].

Our findings revealed that fish was the optimal source of collagen for improving skin hydration. A previous study indicated that collagens sourced from fish skins have diverse amino acid compositions than mammalian collagens [57]. Another study estimated that the yields of collagen derived from fish skin were 50%, collagen derived from fish bones were 40%, and collagen derived from fish fin were 36.4% [58]. Notably, marine collagen and collagen peptides have high bioavailability, potency, and a favorable safety profile [59].

In our investigations, only one study by Schwartz (2019) investigated the effect of collagen sourced from chicken, which was the least among all included studies. However, in the study by Cao et al. on the effects of the oral intake of CPs derived from chicken bones in mice showed that the concentration of moisture in the skin of mice treated with CPs was significantly higher compared with that of the control mice (*p* < 0.05) [55]. Schwastz et al. administered 1 g of collagen from hydrolyzed chicken sternal cartilage daily for 12 weeks to all human participants. The skin hydration of the participants significantly increased by 12.5% (*p* = 0.003) between weeks 6 and 12 [36]. Additionally, it is unclear whether the results can be generalized to the wider population, as the studies were conducted on mice and humans with different characteristics and may not reflect the general population.

### 4.2. Elasticity

Fibril-forming type I collagen is the major collagen in the skin, comprising 90% of the total collagen, and plays a role in structural organization, integrity, and strength and skin [60]. The elastic fiber network imparts elasticity and resilience to the tissues and comprises elastin and microfibrils, which are composed of various proteins [61]. The elasticity of the skin depends on the function of the network, and its formation is a complex process involving many factors. One study showed that the intake of HC downregulated placenta growth factor-2, insulin-like growth factor binding protein 2, insulin-like growth factor binding protein 3, platelet factor 4, serpin E1, and transforming growth factor β-1, and increased type I collagen mRNA and protein levels [62].

Our findings revealed that supplementation with oral collagen improves skin elasticity, which are consistent with previous findings. De Luca et al. found that patients taking marine collagen peptides significantly improved skin elasticity (*p* < 0.0001) [63]. Maia Campos et al. demonstrated that a group treated with oral collagen showed significant differences in the mechanical properties of the skin compared with the baseline and placebo groups after 90 days of treatment only in the net elasticity parameter in the periorbital region [64]. Lee et al. showed that 12 weeks of oral collagen film consumption significantly increased the elasticity of the skin surface (R2), yielding 0.66 ± 0.05 before use to 0.75 ± 0.04 after 12 weeks (*p* < 0.05) [65]. The study conducted by Sone et al. (2018) was conducted on chronologically aged mice, which showed that oral administration of collagen peptides derived from bovine bone can improve the laxity of chronologically aged skin in mice by increasing the skin collagen content and ratio of type I to type III collagen. The study also suggested that collagen peptides may increase antioxidant properties in the body, and proline intake can improve the elasticity of chronologically aged skin in mice [66].

Among the included studies, Yoon et al. showed that in humans, 12 weeks of supplementation with oral collagen significantly improved skin elasticity (3.25, 95% CI 2.33, 4.18) compared with other durations. This finding is consistent with that of an open, blinded, and noncomparative study, which showed 38.31% of improvement in elasticity after consuming oral collagen for 3 months [67]. Another study examined obvious characteristics of skin aging in nude mice after combining treatment with D-galactose and ultraviolet radiation. However, after the oral administration of CP, the concentrations of skin collagen and elastin increased [68]. While studies suggest that oral collagen supplementation may improve skin elasticity, it is important to consider the limitations of the research. The studies used different durations and forms of collagen supplementation, making it difficult to compare the results. Furthermore, the sample sizes of the studies were relatively small, and the human studies relied on self-reported measures of skin elasticity. Additionally, the study on nude mice may not accurately reflect the effects of oral collagen supplementation in humans.

### 4.3. Mechanism

Protein hydrolysates are easier to digest and absorb than intact proteins, which increase the production of amino acids after meals [69]. An in vivo mouse model study found transient increases in the Gly-Pro-Hyp levels in the blood of both humans and mice and that other collagen peptides were also transported to the skin after the ingestion of HC [70]. Kamiyama et al. used [14C] Gly-Pro-Hyp as a tracer for the tripeptide and compared its absorption with 14C-labeled proline in rats. At 14 days after the administration of [14C] Gly-Pro-Hyp, almost all radioactivity disappeared from the organs, except for the skin, with a radioactivity of 70% observed after 6 h [71]. Another similar study observed radioactivity after a single administration of [14C] Gly-Pro-Hyp in the connective tissues including the bones and skin within 24 h [72].

### 4.4. Sensitivity Analysis

In this study, two included RCTs, namely Campos et al. [29] (2.17, 95% CI 1.52, 2.81) Choi et al. [32] (1.61, 95% CI 0.44, 2.78), yielded favorable effects of oral collagen supplementation on skin elasticity. Campos (2015) used a mixture of 10 g of collagen and vitamin A, C, E, zinc as well as excipients, which had beneficial effects, possibly because of its synergism with collagen. A study found that vitamin C triggers a considerable thickening of the epidermis, induces the production of collagen and the formation of elastic microfibrils [73]. By contrast, vitamin A maintains the health of the epithelial cells on the surface of the skin and increases the production of collagen and the extracellular matrix [74,75]. However, because Choi (2014) enrolled participants aged 30–48 years, which were younger than the participants in the other included studies, it is possible that this study yielded better results due to factors such as a potentially lower prevalence of underlying health conditions or greater overall health among the younger participants. This might thus explain why this study yielded better results. A clinical study that contributed that the composition of the basement membrane changed with age showed that the concentrations of collagen IV, collagen IV, and collagen XII decreased over time [76]. Thus, a sensitivity analysis was performed to assess the influence of these two studies, and the results of the corresponding forest plots are provided in the Supplementary Materials. The exclusion of this study resulted in no significant change, and the effects of collagen supplementation remained favorable.

### 4.5. Limitations

This study had several limitations. First, the interventions used in the included studies exhibited some heterogeneity, primarily because of the distinct measurement units and composition of the supplementation. Second, the number of patients included in some studies was less than 40. Therefore, a small sample size may have resulted in a slight RoB. Third, the patients’ lifestyle habits were not included in the analysis. For example, HC supplementation in patients with healthier lifestyle habits could have presented more evident results in improving the appearance of the skin. Thus, additional studies, specifically large clinical trials, are needed.

## 5. Conclusions

The findings of this study revealed that HC supplementation can improve skin hydration and elasticity. In addition, the long-term use of collagen yields more favorable effects on skin hydration and elasticity than the short-term use of collagen. Nevertheless, large-scale randomized control trials are required to examine the clinical benefits of oral collagen supplements.

## Data Availability

Data will be made available on reasonable request.

## Supplementary Materials

The following supporting information can be downloaded at: https://www.mdpi.com/article/10.3390/nu15092080/s1, Figure S1. Elasticity-sensitivity analysis; Figure S2. Hydration-sensitivity analysis.
