# Supplementary figures and images for: Effects of Oral Collagen for Skin Anti-Aging: A Systematic Review and Meta-Analysis
Source: Nutrients. 2023 Apr 26;15(9):2080. doi: 10.3390/nu15092080 (PMC10180699; doi:10.3390/nu15092080)

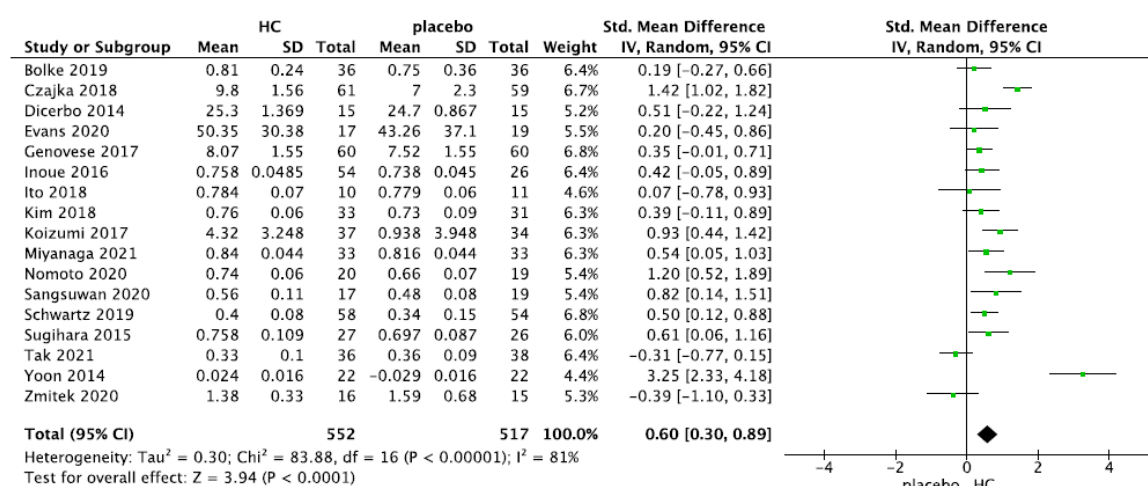

Figure S1. Elasticity-sensitivity analysis. [26–28, 31,33-35,37,39–44,47–49]

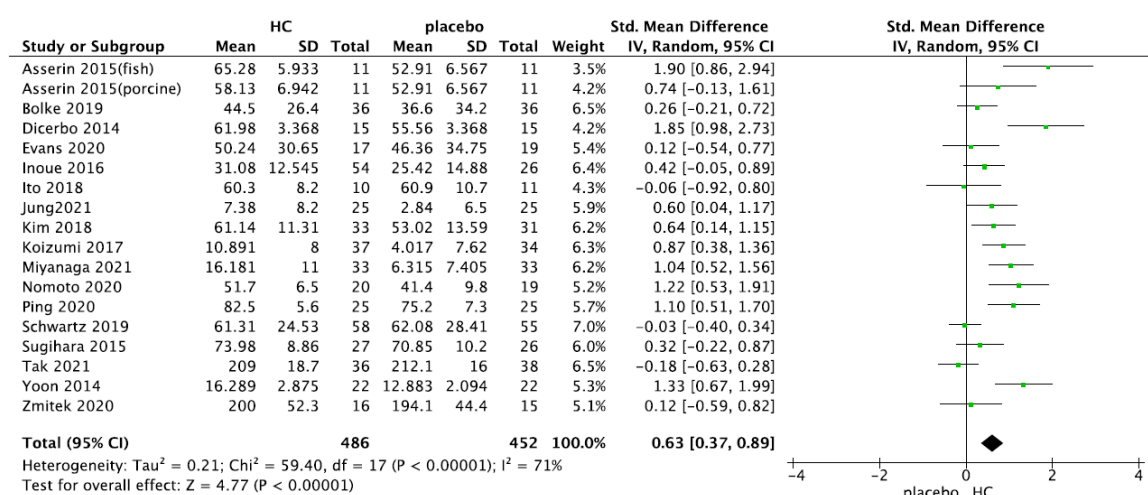

Figure S2. Hydration-sensitivity analysis. [26–28,30–31,33-35,39,40,43,44,46–50]

Supplement: Supplementary file 1 [file nutrients-15-02080-s001.zip › nutrients-2311071 supplementary.pdf]
